# Actinomycetes associated with hymenopteran insects: a promising source of bioactive natural products

**DOI:** 10.3389/fmicb.2024.1303010

**Published:** 2024-02-28

**Authors:** Umar Diarra, Tamara Osborne-Naikatini, Ramesh Subramani

**Affiliations:** School of Agriculture, Geography, Environment, Ocean and Natural Sciences, The University of the South Pacific, Suva, Fiji

**Keywords:** actinomycetes, hymenoptera, symbiosis, natural products, bioactive compounds

## Abstract

In recent years, the insect microbiome has become the focus of many actinomycete researchers in their search for novel bioactive compounds with members of the order Hymenoptera at the forefront of the revolution. Hymenoptera encompasses all bees, wasps, ants, and sawflies and is the third largest insect order by species richness. Additionally, Hymenoptera is the most diverse insect order in terms of ecological roles, behaviors, and social systems, thus making it an ideal starting point in the search for symbiotic actinomycetes. The aim of this review is to summarize current knowledge on hymenopteran associations with actinomycetes including information on interactions between actinomycetes and hymenopterans, isolation, and screening methodologies, as well as novel actinomycete species and natural products discovered between early 2013 and 2023. A total of 19 new species were discovered within this time period, with the genus *Streptomyces* being represented by 11 species while the remaining 8 belonged to rare actinomycetes genera. In addition, 35 novel compounds were reported from hymenopteran-associated actinomycetes within the same time period with the majority originating from *Streptomyces* strains. The reported novel compounds exhibit a range of biological activities including antibacterial, antifungal, anticancer, anti-enzymatic, and antiproliferative activity, as well as cytotoxicity.

## Graphical Abstract

Metabolites of hymenopteran-associated actinomycetes (created using BioRender.com). 
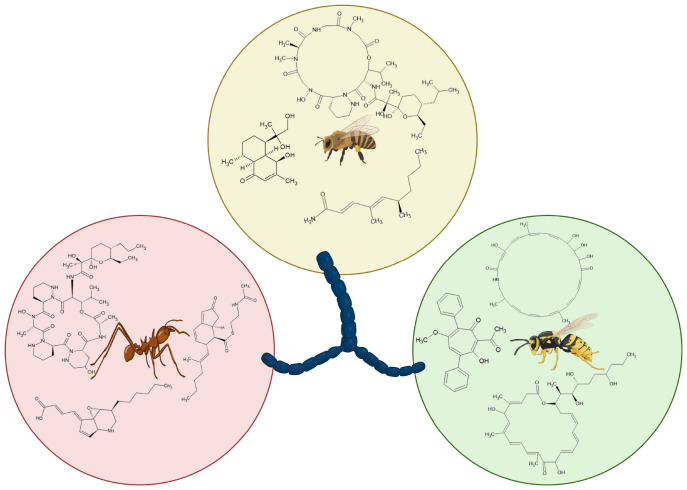

## 1 Introduction

Among the most alarming health concerns in the 21st century, is the issue of antimicrobial resistance. Antimicrobial resistance (AMR) occurs in bacteria, fungi, viruses, and parasites, but bacteria are the most concerning organisms due to the frequency of occurrence of resistance (Prestinaci et al., 2015). Currently, AMR is worse than ever with all known antibiotic classes having corresponding

resistant organisms. To understand the magnitude of the problem, the US-CDC’s 2013 annual report on AMR lists three resistant human pathogens (*Clostridium difficile*, Carbapenem-resistant Enterobacteriaceae, and Drug-resistant *Neisseria gonorrhoeae*) as urgent threats (CDC, 2013). In comparison, the latest report, which was published in 2019, included two additional pathogens (Carbapenem-resistant *Acinetobacter* and *Candida auris*) in the category (CDC, 2019). Furthermore, more than 2.8 million cases of AMR infections and about 36,000 deaths are reported annually in the US alone (CDC, 2019; Kadri, 2020). In addition to the immense loss of lives, the current AMR crisis has placed a heavy burden on the global economy.

Nature has long been a source of inspiration and innovation in the realm of drug discovery and development. From the rainforests to the depths of the oceans, organisms have gifted us with an array of bioactive compounds. Among the diverse organisms that have caught the attention of scientists are microorganisms known as actinomycetes which have gifted mankind with some of the most important antibiotics. Actinomycetes, in this review, refers to members of class actinomycetes of phylum Actinomycetota and comprises of Gram-positive filamentous bacteria with a high Guanine-plus-Cytosine (G+C) content in their genomes. Actinomycetes have long been recognized as prolific producers of natural products, including antibiotics, anticancer agents, immunosuppressants, and enzymes etc (Barka et al., 2015). Their antibiotic production ability is unrivaled with actinomycete-derived antibiotics accounting for 70% of clinically used antibiotics (Subramani and Sipkema, 2019). Traditionally, actinomycetes were isolated from soil and marine environments, which have served as the primary reservoirs for drug discovery. However, diminishing returns in metabolite discovery in the past few decades have spurred scientists to not only revise their methods but also explore alternative reservoirs for actinomycetes (Matsumoto and Takahashi, 2017; Jose et al., 2021). In recent years, a growing body of research has unveiled the tremendous potential of symbiotic actinomycetes, with hymenopteran insects emerging as a particularly promising host group (Poulsen et al., 2011; Nechitaylo et al., 2014; Matarrita-Carranza et al., 2017; Chevrette et al., 2019; Long et al., 2022). Hence, this review focuses on associations between actinomycetes and hymenopteran insects as well as bioactive compounds produced.

## 2 Insect-microbe symbioses

Being ubiquitous in nature, microorganisms have developed diverse and unique interactions with a wide range of host organisms, spanning several eukaryotic phyla. Insects are no exception to this rule as the average healthy insect is said to contain more microbial cells than insect cells (Douglas, 2015). Nonetheless, among the multitude of microbial cells that may be present in or on an insect at any given time, very few are symbiotes of the insect. Due to the anatomy and physiology of insects, different regions of the body differ in the ease of invasion by microbes. Regions such as the gut and cuticle are considered open and thus, susceptible to colonization by microorganisms. As such, these regions are dominated by free-living microbes which in a sense, utilize their insect host as a means of dispersal, and microbes that are common among insects and other animals but are rarely isolated from environmental samples (Douglas, 2015). However, several insects have evolved specialized structures to house specific groups of microbes. For example, cuticular structures such as mycetangia in ambrosia beetles are used to store mutualistic fungi which the beetles cultivate as a food source and similarly, antennal gland reservoirs in beewolf wasps and foveae in attine ants are used as culture vessels for mutualistic bacteria of the order Actinomycetales (Currie et al., 2006; Li et al., 2018). These modified structures are not exclusive to the cuticle as the guts of some insect species are also known to exhibit modifications. The midguts of many heteropteran insects for example, are known to contain evaginations known as crypts or caeca which are inhabited by symbiotic bacteria (Engel and Moran, 2013). *Ishikawaella capsulata*, a mutualistic symbiote of the Japanese stinkbug *Megacopta punctatissima*, has been shown to inhabit crypts in the midgut region of its host (Fukatsu and Hosokawa, 2002).

Microorganisms that are capable of breaching open regions gain access to less accessible (closed) regions of the insect body. An estimated 10–20% of insect species possess specialized cells, known as bacteriocytes, which mainly function in housing and maintaining symbiotic bacteria (Douglas, 2011). A variation of the bacteriocyte, known as the mycetocyte, is known to contain fungi such as yeasts in some insect species. In addition, gut bacteria can gain access to the hemolymph (which is rich in nutrients) provided they are able to tolerate the conditions (e.g., pH, oxygen content) and bypass the insects’ immune system. Bacteria in tissues such as the hemolymph are usually associated with infections, for example the nematode *Steinernema carpocapsae* is involved in a mutualistic relationship with the bacterium *Xenorhabdus nematophila* in which *S. carpocapsae* invades the gut of an insect larva and subsequently releases *X. nematophila* at the homocoel (i.e., into the hemolymph) where it compromises the host’s immune system (Singh et al., 2015). Nonetheless, hemolymph-associated mutualistic bacteria have also been reported in some insects. According to Tufts and Bextine (2009), *Bacillus cereus* and *B. thuringiensis* are prominent in the hemolymph of imported fire ants (*Solenopsis invicta*) and the latter is thought to produce toxins that aid in the ants’ defense.

## 3 Actinomycetes as symbiotes

While the majority of actinomycetes exist as free-living cells in soil and water, several actinomycetes have developed associations host organisms such as fungi, plants, and animals. Actinomycete interactions are often mutualistic or commensal in nature, but in rare cases, can be parasitic. In plants, for example, symbiotic actinomycetes of the genera *Frankia* and *Micromonospora* form mycorrhizal associations, aiding in nitrogen fixation in exchange for nutrients (Carro et al., 2013). On the contrary, *Streptomyces* spp. such as *S. scabies*, *S. aureofaciens*, *S. acidiscabies*, and *S. ipomoeae*, are associated with scab and rot diseases in potatoes, beets, and carrots (Locci, 1994).

### 3.1 Actinomycetes in the insect world

Currently, most known mutualisms between Actinomycetota and insects involve members of the class Actinomycetia (Seipke et al., 2012; van der Meij et al., 2017; Baranova et al., 2022). Thus far, actinomycetes have been isolated from insects of the Orders Blattodea, Coleoptera, Dermaptera, Hymenoptera, Lepidoptera, Orthoptera, and Trichoptera (Van Moll et al., 2021; Baranova et al., 2022). Symbiotic associations between actinomycetes and insects are often mutualistic, with both partners contributing toward the wellbeing of each other. The insect host is often tasked with providing a suitable shelter and nutrients to support the growth and reproduction of its symbiote. For example, in leaf-cutter ants, cuticular invaginations on the thorax and legs serve as culture vessels and stores for *Pseudonocardia* symbiotes whereas antennal gland reservoirs serve the same purpose for the *Streptomyces* symbiotes of Beewolf wasps (Douglas, 2015). The host insect is also responsible for the transmission of its symbiote either horizontally or vertically, with the latter being often restricted to obligate symbioses. On the other hand, actinomycetes may provide nutritional benefits to their insect hosts as in the case of hemipteran insects. Studies have demonstrated that *Rhodnius prolixus* and *Triatoma infestans*, which are both vectors of chagas disease, are nutritionally dependent on actinomycetes of the genera *Rhodococcus* and *Corynebacterium* (Kaltenpoth, 2009). Nonetheless, defense is the most well-known and studied service provided by actinomycetes for their insect hosts. This comes as no surprise as actinomycetes are the most prolific producers of antibiotics among microbes. Furthermore, besides producing antagonistic compounds that directly inhibit parasites of their hosts, actinomycetes may also contribute to the host’s defense indirectly. Studies of bacterial-insect mutualisms have identified two main mechanisms of indirect defense; (1) the occupation of susceptible niches in and on the host to outcompete parasites (Koehler, 2014), and (2) the modulation of the insect’s innate immunity (Koehler, 2014; Douglas, 2015).

## 4 The order Hymenoptera

Hymenoptera comprises more than 150,000 described species, making it the third largest insect order in terms of species richness. Hymenoptera consists of Suborder Symphyta which includes sawflies, horntails, and parasitic wood wasps and Suborder Apocrita which comprises all ant, wasp, and bee species. Along with termites (Order Blattodea), many hymenopterans exhibit eusocialism, a system characterized by the presence of reproductive division of labor, brood care, and an overlap of generation of individuals of the same colony (Matarrita-Carranza et al., 2017). At the same time, solitary species of hymenopterans are also well-known and studied. Hymenopterans are holometabolous insects, meaning they undergo complete metamorphosis (i.e., from egg to larva, larva to pupa and pupa to adult). Furthermore, brood care is common among social hymenopterans while solitary species usually exhibit parasitoidism. Dietary habits are also diverse within Hymenoptera and range from carnivory (insectivory and in rare cases, cannibalism) to herbivory (nectarivory, palynivory, granivory, and frugivory). Furthermore, dietary habits often vary for the different developmental stages (Hyodo et al., 2011; Maák et al., 2020). In some ants for example, larval diets tend to be rich in proteins whereas adult workers require sugar-rich diets (Pohl et al., 2016). These vast variations in lifestyle within the Hymenoptera make it an ideal starting point in the search for novel actinomycetes, unique symbioses and consequently, new bioactive natural products.

### 4.1 Hymenopteran-actinomycete symbioses

With regards to symbioses with actinomycetes, few insect groups have been studied as extensively as the Order Hymenoptera (Van Moll et al., 2021; Baranova et al., 2022). Hymenopteran-actinomycete symbioses are predominantly defensive in nature and often follow either of two mechanisms: (1) the protection of the host’s nutritional resources or, (2) the protection of the host and its offspring.

#### 4.1.1 Protection of the host’s nutritional resources

The richness in species of the Order Hymenoptera means that species are often forced to compete over limited resources especially with regards to nutrition (Houadria et al., 2015). Possibly in response to this pressure, certain lineages of hymenopterans have carved out specific nutritional niches in their respective environments. One important adaptation is the development of farming, ranging from plant farming in plant-ants to fungus-farming in attine ants (Chomicki et al., 2019; Shik et al., 2021). While an effective strategy to reduce inter-species competition, farming presents unique challenges, the most prominent of which is parasitism. Nest parasites are generally common in hymenopterans particularly in soil-nesting and social species (Wcislo, 1996; Schmid-Hempel, 2019). Nonetheless, most nest parasites are generalists and thus, can be controlled via behavioral adaptations such as grooming and weeding. Specific lineages of parasites, however, are specialized in infecting nutritional resources, essentially competing with hymenopterans over their food (Nechitaylo et al., 2014; Gotting et al., 2022). It is now known that certain farming hymenopterans recruit actinomycetes for defense (Kaltenpoth et al., 2005; Batey et al., 2020). This defensive function, in all studied cases, is mediated by individual compounds or mixtures produced by the symbiotic actinomycetes (Oh et al., 2009; Fukuda et al., 2021).

##### 4.1.1.1 Attine ant-*Pseudonocardia* mutualism

Ants of the tribe Attini (Subfamily Myrmicinae, Family Formicidae) are among a handful of insects that are extensively known to cultivate fungi for food (Goldstein and Klassen, 2020). The most primitive form of agriculture emerged in lower attines about 55–65 million years ago and subsequently diversified into other systems (Li et al., 2018; Mueller et al., 2018). Lower agriculture is practiced by members of the basal groups in the Palaeoattini and characteristically involves the cultivation of Basidiomycetes from two distinct clades (Clade 1 and 2) of the Leucocoprineae tribe (Family Agaricaceae) (Mehdiabadi et al., 2012; Mueller et al., 2018). Still within the Paleoattini, a few members of the genus *Apterostigma* have specialized in the cultivation of the phylogenetically distant coral fungi (Family Pterulaceae) (Cafaro et al., 2011; Douglas, 2022). Ants of the genus *Cyphomyrmex* (Subfamily Neoattini) are known to cultivate a single clade of single-celled dimorphic leucocoprineaceous fungi (Yeast agriculture) (Douglas, 2022). Finally, higher agriculture encompasses the agricultural systems of the ant genera; *Atta*, *Acromyrmex*, *Trachymyrmex*, and *Sericomyrmex* which predominantly cultivate fungi from two clades (clade A and B) of the Leucocoprineae tribe (Solomon et al., 2011; Mueller et al., 2018). *Trachymyrmex* spp. have also been found to cultivate lower-attine fungal cultivars (Mueller et al., 2018). The system of fungiculture practiced by *Atta* and *Acromyrmex* species slightly differs from traditional higher agriculture as members of the genera exclusively cultivate fungi on freshly cut leaves as opposed to dead plant material and as such, is appropriately termed leaf-cutter agriculture (Cafaro et al., 2011; Li et al., 2018). Leaf-cutter ants predominantly cultivate *Leucocoprinus gongylophorus* which is thought to be an obligate symbiote of higher attine ants due to its; (i) strict vertical transmission, (ii) absence in the free-living condition, and (iii) production of nutrient-rich hyphal swellings known as gongylidia that are absent in all other Leucocoprineae (Weber, 1972; Solomon et al., 2011; Mehdiabadi et al., 2012).

**FIGURE 1:**
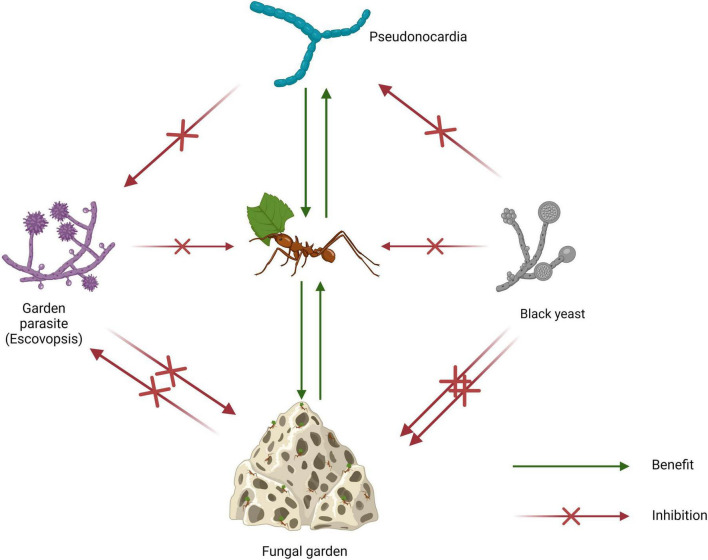
Schematic diagram of the attine ant symbiosis showing interactions between symbiotes (created using BioRender.com).

Fungi from the genus *Escovopsis* have specialized in infecting attine ant fungal cultivars and represent the most significant threats to the ants’ food resources (Figure 1; Meirelles et al., 2014; Jiménez-Gómez et al., 2021; Montoya et al., 2021). The fungal garden parasites have attained a level of specialization that is suggestive of co-evolution alongside the ants and their fungal cultivars (Mueller et al., 2018; Douglas, 2022). In response to *Escovopsis* infections, attine ants have developed adaptations such as fungus grooming, weeding and general nest hygiene as well as the use of metapleural gland secretions (Currie et al., 1999; Currie and Stuart, 2001; Fernández-Marín et al., 2009). Furthermore, some leafcutter fungal cultivars have been shown to be capable of inhibiting *Escovopsis* associated with lower attines (Birnbaum and Gerardo, 2016). Nonetheless, none of these behavioral and chemical adaptations have proven as effective as the recruitment of mutualistic actinomycetes of the genus *Pseudonocardia* as biocontrol agents (Figure 1; Goldstein and Klassen, 2020). Depending on the attine ant species, *Pseudonocardia* symbiotes are localized on different regions and/or structures of the ants’ body. In ants of the genus *Apterostigma*, *Pseudonocardia* are contained on the ventral surface of the propleura or metapleural whereas in ants of the genus *Trachymyrmex*, the symbiotes occur on tubercles which are contained within caeca (Cafaro et al., 2011; Caldera and Currie, 2012). However, in both cases, the area of localization of the *Pseudonocardia* symbiote is lined by pores associated with exocrine gland cells which are thought to play the role of nourishing the bacterial symbiote (Poulsen et al., 2003). This ability to control nutrient availability, by extension, allows attine ants to regulate the growth of the symbiote as per demand (Currie et al., 2003; Goldstein and Klassen, 2020).

The effectiveness of *Pseudonocardia* as biological control for *Escovopsis* has been demonstrated *in vitro* through antagonism experiments and *in vivo* within the context of the attine ant mutualism (Currie et al., 1999; Little and Currie, 2008; Poulsen et al., 2010; Meirelles et al., 2014; Dângelo et al., 2016). There is also evidence to support the vertical transmission and specificity of *Pseudonocardia* symbiotes among attine ant species (Cafaro et al., 2011; Andersen et al., 2013). Furthermore, *Pseudonocardia* spp. associated with *Trachymyrmex septentrionalis* have been shown to be capable of selectively inhibiting other attine ant associated *Pseudonocardia* using a thiopeptide antibiotic (GE37468) likely acquired from soil bacteria (Chang et al., 2020). Early studies of attine-ant associations postulated the idea that *Pseudonocardia* are locked in an evolutionary arms race with *Escovopsis* and that both organisms have been coevolving. However, a reevaluation of this evidence by Mueller (2012) bred an opposing viewpoint which suggests that attine ants may utilize a community of bacteria for garden defense and that non-*Pseudonocardia* isolated from attine ant biofilms may not be contaminants as previously thought. Furthermore, the author argues that *Pseudonocardia* are not specialized in the inhibition of *Escovopsis* and are capable of inhibiting a much broader spectrum of pathogens (Mueller, 2012).

Even more fascinating is the fact that black yeasts of the genus *Phialophora* and associates (Ascomycota: Chaetothyriales) have been reported to be associated with the attine ant symbiosis (Figure 1; Little and Currie, 2007; Bizarria et al., 2022). Black yeasts are parasites known to derive nutrients from and suppress the growth of attine-ant *Pseudonocardia* (Little and Currie, 2007). Little and Currie (2008) demonstrated, using infection experiments, that *Apterostigma pilosum* colonies infected with black yeast are significantly less effective at defending their fungal gardens than uninfected colonies.

Interactions in the complex web that is the attine symbiosis are facilitated by secondary metabolites produced by participating organisms. Several compounds with bioactive properties have been reported from the attine ant symbiosis. Recently, a study of a *Trachymyrmex* sp. in Brazil identified two new compounds, conocandin B and dentigerumycin F, produced by *Escovopsis* and *Pseudonocardia*, respectively. Interestingly, conocandin B was found to upregulate the production of dentigerumycin F and vice versa (Bae et al., 2021).

#### 4.1.2 Protection of the host and its offspring

Pathogens do not only contend over a host’s nutritional resources as the host organism itself can be a source of nutrients for specialized pathogens. For example, the fungus *Ophiocordyceps unilateralis* is known to hijack ant hosts for its reproduction and dispersal (Evans et al., 2011). Similarly, American foulbrood is a disease in honey bee larvae caused by the bacterium *Paenibacillus larvae* (Stephan et al., 2020). Actinomycetes, due to their prevalence in hymenopterans and potential as antimicrobial producers, are good candidates for the defense of hosts.

##### 4.1.2.1 Beewolf wasp-*Streptomyces* symbiosis

Beewolf wasps or bee-killer wasps are solitary wasps of the tribe Philanthini (Family Crabonidae) that nest in sandy soil and, as their name suggests, primarily prey on honeybees (Kaltenpoth et al., 2005; Nechitaylo et al., 2014). Nests are constructed by female beewolves to house developing larvae and their food, usually over the winter. In summer, fully developed adults emerge from the burrow nests to continue the cycle (Seipke et al., 2012). As such, it makes sense that nest hygiene is very critical for the survival of young beewolves, especially considering that the physical (warmth and high humidity) and biological (exposure of larvae to dead insects) conditions within burrow nests favor the growth of parasites (Kaltenpoth et al., 2012; Koehler et al., 2013). Like fungus-farming ants, beewolf wasps have developed adaptations to manage parasites (Kaltenpoth et al., 2012; Seipke et al., 2012; Nechitaylo et al., 2014; Goettler et al., 2022). One of such adaptations is the use of cephalic-gland secretions to embalm paralyzed prey as food for the developing offspring (Kaltenpoth et al., 2005). Recent reports also show that beewolves emit large amounts of gaseous nitric oxide to protect themselves and their food from pathogenic fungi (Strohm et al., 2019). Additionally, beewolf wasps have also acquired an actinomycete symbiote to aid in defense (Kroiss et al., 2010). Female beewolf wasps inoculate brood cells thoroughly with the whitish-appearing symbiotic actinomycete prior to oviposition. After oviposition and before the commencement of pupation, beewolf larvae inoculate the actinomycete on their cocoon. Kaltenpoth et al. (2012) demonstrated experimentally that the symbiotic actinomycete of beewolf wasps significantly decreases larval mortality during development. The results of the study showed that only one out of 15 (6.7%) larvae without access to the symbiotic actinomycete survived till emergence. In contrast, there was an 83.3% (15/18) chance of emergence in the control group (i.e., larvae with access to the symbiote). Furthermore, in addition to its defensive role, the actinomycete symbiote of beewolves is also believed to serve as a directional cue for larvae during emergence from cocoons (Kaltenpoth et al., 2012; Goettler et al., 2022).

**FIGURE 2:**
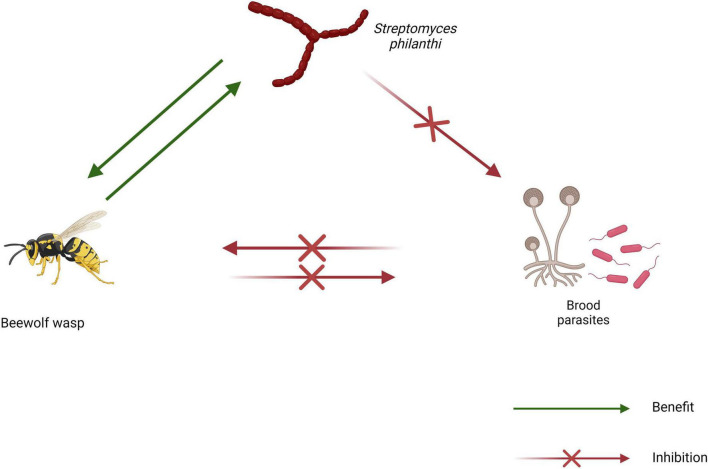
Schematic diagram of the beewolf wasp symbiosis showing interactions between symbiotes (created using BioRender.com).

The Philanthini tribe comprises three genera; *Trachypus*, *Philanthus*, and *Philanthinus*, all of which have been found to associate with different biovars of the actinomycete *Streptomyces philanthi* (Figure 2; Nechitaylo et al., 2014). In all three genera, the *Streptomyces* symbiote is cultivated and maintained in specialized cuticular invaginations on the antennae (Figure 2; Kaltenpoth et al., 2012; Nechitaylo et al., 2014). These antennal reservoirs are presumably connected to the hemolymph and gland cells through which the *Streptomyces* symbiotes are thought to obtain nourishment from the host (Kaltenpoth et al., 2012). Evidence strongly suggests that *Streptomyces* symbiotes are transmitted vertically in beewolf wasps. Larvae of the species have been observed ingesting the symbiote from inoculated nests prior to pupation (Kaltenpoth et al., 2005). The same study also demonstrated a general lack of interest in nest construction by an adult female wasp lacking the symbiote.

The defensive function of *S. philanthi* in beewolf wasps is believed to be mediated by a cocktail of antibiotics. Analysis of methanol extracts of *Philanthus triangulum* cocoons revealed that streptochlorin, piericidin A1, and piericidin B1 were the three most abundant compounds produced by *Streptomyces philanthi* (Engl et al., 2018). Additionally, the total amount of these three compounds was shown to steadily increase in the 2 weeks following inoculation and persist up till the emergence of the offspring. Furthermore, Boukaew et al. (2013) reported that wheat seed cultures inoculated with *S. philanthi* RM-1-138 contained up to 36 volatile organic compounds, of which four (dimethyl disulfide, dimethyl trisulfide, geosmin, and benzene ethanol) have been reported to possess antimicrobial properties. These volatile compounds could control rice sheath blight disease, having suppressed the growth of plant pathogenic fungi such as *Rhizoctonia solani* PTRRC-9, *Pyricularia grisea* PTRRC-18, *Bipolaris oryzae* PTRRC-36 and *Fusarium fujikuroi* PTRRC-16 *in vitro* (Boukaew et al., 2013). The same *S. philanthi* strain was later shown to protect soybean seeds from aflatoxin-producing fungi and contain additional compounds such as l-linalool, 2-mercaptoethanol and heneicosane (Boukaew and Prasertsan, 2020).

### 4.2 Other studied hymenopterans associated with actinomycetes

#### 4.2.1 Family Formicidae: subfamily Formicinae

Formicinae is one of the most diverse ant subfamilies, comprising about 3,030 species widely distributed around the world (Ward et al., 2016).

##### 4.2.1.1 Genus *Camponotus*

Carpenter ants (genus *Camponotus*) are known for their unique ability to excavate galleries within wood to create their nests. Unlike termites, carpenter ants are unable to digest the polymers in wood due to the absence of specialized microbes in their midguts (Brune, 2014). However, carpenter ants are known to be involved in a symbiosis with bacteria of the genus *Blochmannia* in which the bacteria reside in the host’s midguts and aid in the synthesis of essential and non-essential amino acids (Zientz et al., 2006). Zakalyukina et al. (2019) described the isolation of a *Streptomyces violaceochromogenes* strain capable of producing nybomycin from *Camponotus vagus*. Another study by the same author demonstrated the cellulose-degrading ability of actinomycetes isolated from *Camponotus vagus* (Zakalyukina et al., 2021). Moreover, novel actinomycete species such as *Amycolatopsis camponoti*, *Microbispora camponoti*, *Nocardia camponoti*, *Streptomyces capitiformicae*, and *Streptomyces camponoti* among others, have been reported from carpenter ants (Table 1). Studied carpenter ant species include *Camponotus vagus*, *C. japonicus* and *C. kiusiuensis*.

**TABLE 1:** Novel species of actinomycetes isolated from hymenopteran insects between January 2013 and May 2023.

Strain/family	Hymenopteran host		Source	Method	Isolation medium	Sampling location	References
	**Family**	**Common name (scientific name)**					
*Amycolatopsis camponoti* sp. nov./ Streptomycetaceae	Formicidae	Carpenter ant (*Camponotus vagus*)	Tissue of adult workers	Five individuals were washed three times using sterile distilled water, after which they were macerated using a tissue microhomogenizer along with sterile saline solution. Subsequently, aliquots of these homogenized samples were plated onto the isolation medium. The plates were then incubated at a temperature of 28 °C for a period of 14 days.	Actinomycete isolation agar (2 g Sodium caseinate, 0.1 g L-Asparagine, 4 g Sodium propionate, 0.5 g K_2_HPO_4_, 0.001 g FeSO_4_x7H_2_O, 0.1 g MgSO_4_x7H_2_O, and 15 g agar per liter of H_2_O) supplemented with nystatin (50 μg/mL) and nalidixic acid (10 μg/mL).	Kasimovsky District, Ryazan region, Russia	Zakalyukina et al., 2022a
*Streptomyces lasii* sp. nov./ Streptomycetaceae	Formicidae	Yellow meadow ant (*Lasius flavus*)	Head	Twenty individuals were surface-sterilized in 70 % ethanol for 60 s and then washed three times in sterile distilled water. Surface-sterilized ants were divided into head, mesosoma, and gaster, and each body part was separately put in 500 μL of sterile water with shaking on a rotary shaker at 180 rpm at 28°C for 30 min. Subsequently, a 200 μL sample of the suspension of the heads was spread on the isolation medium.	Chitin agar (3 g chitin, 0.575 g K_2_HPO_4_, 0.183 g MgSO_4_x7H_2_O, 0.275 g KH_2_PO_4_, 0.0075 g FeSO_4_x7H_2_O, 0.00075 g MnCl_2_x4H_2_O, 0.00075 g ZnSO_4_x7H_2_O, and 15 g agar per 750 mL of H_2_O) supplemented with cycloheximide (50 mg/L) and nalidixic acid (20 mg/L)	Formicary at Northeast Agriculture University, Harbin, China	Liu et al., 2018
*Streptomyces capitiformicae* sp. nov./ Streptomycetaceae	Formicidae	Japanese Carpenter ant (*Camponotus japonicus*)	Head	Same as (Liu et al., 2018)	Sodium succinate-asparagine agar (0.2 g asparagine, 1 g sodium succinate, 0.2 g CaCl_2_x2H_2_O, 0.001 g FeSO_4_x7H_2_O, 0.3 g KCl, 0.9 g KH_2_PO_4_, 0.6 g K_2_HPO_4_x3H_2_O, and 20 g agar per liter of H_2_O; pH-7.2) supplemented with cycloheximide (50 mg/L) and nalidixic acid (20 mg/L)	Formicary at Northeast Agriculture University, Harbin, China	Jiang et al., 2018
*Streptomyces amphotericinicus* sp. nov./ Streptomycetaceae	Formicidae	Japanese carpenter ant (*Camponotus Japonicus*)	Head	Same as (Liu et al., 2018)	Sodium succinate-asparagine agar supplemented with cycloheximide (50 mg/L) and nalidixic acid (20 mg/L)	Formicary at Northeast Agriculture University, Harbin, China	Cao et al., 2017
*Virgisporangium myanmarense* sp. nov./ *Micromonosporaceae*	Unknown	Unspecified ant	Anthill soil	0.5 g of air-dried anthill soil was placed in a beaker (46 mm in diameter and 60 mm deep), which was then gently flooded with 50 ml of 10 mM-phosphate buffer containing 10% soil extract. The vessel was loosely capped with aluminium foil and incubated statically at 30°C for 90 min to allow for liberation of motile zoospores. An 8 ml portion of the flooding solution was then transferred into a screw-cap test tube (16.5 × 105 mm) and centrifuged (room temperature, 20 min, 1,500 × *g*) in a swinging bucket rotor. After the tube was allowed to settle for 30 min, a portion of the supernatant containing zoospores was serially diluted with sterile tap water, and 0.2 ml aliquots of this dilution were plated in triplicate onto the isolation medium	Humic acid-vitamin agar (1.0 g Humic acid, 0.5 g Na_2_HPO_4_, 1.71 g KCl, 0.05 g MgSO_4_x7H_2_O, 0.01 g FeSO_4_x7H_2_O, 0.02 g CaCO_3_, and 18 g agar per liter of H_2_O; pH of 7.2) supplemented with cycloheximide (50 mg/L) and nalidixic acid (20 mg/L)	Anthill soil sample located at Bagan, Myanmar	Yamamura et al., 2017
*Streptomyces lasiicapitis* sp. nov./ Streptomycetaceae	Formicidae	Jet black ant (*Lasius fuliginosus*)	Head	Same as (Liu et al., 2018)	Humic acid-vitamin agar supplemented with cycloheximide (50 mg/L) and nalidixic acid (20 mg/L)	Formicary at Northeast Agriculture University, Harbin, China	Ye et al., 2017
*Streptomyces camponoti* sp. nov./ Streptomycetaceae	Formicidae	Japanese carpenter ant (*Camponotus japonicus*)	Cuticle	Same as (Liu et al., 2018)	Gause’s synthetic agar no. 1 (0.01 g FeSO_4_x7H_2_O, 0.5 g MgSO_4_x7H_2_O, 0.5 g NaCl, 0.5 g K_2_HPO_4_, 1 g KNO_3_, and 15 g agar per liter of distilled water; pH-7.4), and Sodium succinate-asparagine agar. Each supplemented with cycloheximide (50 mg/L) and nalidixic acid (20 mg/L)	Formicary at Northeast Agriculture University, Harbin, China	Piao et al., 2017
*Streptomyces cuticulae* sp. nov./ Streptomycetaceae	Formicidae	Japanese carpenter ant (*Camponotus japonicus*)	Cuticle	Same as (Liu et al., 2018)	Gause’s synthetic agar no. 1 supplemented with cycloheximide (50 mg/L) and nalidixic acid (20 mg/L)	Formicary at Northeast Agriculture University, Harbin, China	Piao et al., 2017
*Microbispora camponoti* sp. nov./ Streptosporangiaceae	Formicidae	Japanese carpenter ant (*Camponotus japonicus*)	Cuticle	Same as (Liu et al., 2018)	Humic acid-vitamin agar supplemented with cycloheximide (50 mg/L) and nalidixic acid (20 mg/L)	Formicary at Northeast Agriculture University, Harbin, China	Han et al., 2016
*Streptomyces camponoticapitis* sp. nov./ Streptomycetaceae	Formicidae	Japanese carpenter ant (*Camponotus japonicus*)	Head	Same as (Liu et al., 2018)	Tap water yeast extract agar (0.25 g yeast extract [Oxoid], 0.5 g of K_2_HPO_4_, and 18 g of agar [Oxoid] per liter of tap water) supplemented with cycloheximide (50 mg/L) and nalidixic acid (20 mg/L)	Formicary at Northeast Agriculture University, Harbin, China	Li et al., 2016
*Nocardia lasii* sp. nov./ Nocardiaceae	Formicidae	Jet black ant (*Lasius fuliginosus*)	Cuticle	Same as (Liu et al., 2018)	Humic acid-vitamin agar supplemented with cycloheximide (50 mg/L) and nalidixic acid (20 mg/L)	Formicary at Northeast Agriculture University, Harbin, China	Liu et al., 2016a
*Actinocorallia lasiicapitis* sp. nov./ Thermomono- sporaceae	Formicidae	Jet black ant (*Lasius fuliginosus*)	Head	Same as (Liu et al., 2018)	Gause’s synthetic agar no. 1 supplemented with cycloheximide (50 mg/L) and nalidixic acid (20 mg/L)	Formicary at Northeast Agriculture University, Harbin, China	Liu et al., 2016c
*Nocardia camponoti* sp. nov./ Nocardiaceae	Formicidae	Japanese carpenter ant (*Camponotus japonicus*)	Head	Same as (Liu et al., 2018)	Humic acid-vitamin agar supplemented with cycloheximide (50 mg/L) and nalidixic acid (20 mg/L)	Formicary at Northeast Agriculture University, Harbin, China	Liu et al., 2016b
*Promicromonospora alba* sp. nov./ Promicromono- sporaceae	Formicidae	Japanese carpenter ant (*Camponotus japonicus*)	Cuticle	Same as (Liu et al., 2018)	Humic acid-vitamin agar supplemented with cycloheximide (50 mg/L) and nalidixic acid (20 mg/L)	Formicary at Northeast Agriculture University, Harbin, China	Guo et al., 2016
*Streptomyces formicae* sp. nov./ Streptomycetaceae	Formicidae	Japanese carpenter ant (*Camponotus japonicus*)	Head	Same as (Liu et al., 2018)	Gause’s synthetic agar no. 1 supplemented with cycloheximide (50 mg/L) and nalidixic acid (20 mg/L)	Formicary at Northeast Agriculture University, Harbin, China	Bai et al., 2016
*Micromonospora polyrhachis* sp. nov./ Micromonosporaceae	Formicidae	Chinese black ant (*Polyrhachis vicina*)	Whole tissue	Same as (Liu et al., 2018)	Humic acid-vitamin agar supplemented with cycloheximide (50 mg/L) and nalidixic acid (20 mg/L)	Formicary at Northeast Agriculture University, Harbin, China	Xiang et al., 2014
*Streptomyces polyrhachii* sp. nov./ Streptomycetaceae	Formicidae	Chinese black ant (*Polyrhachis vicina*)	Whole tissue	Same as (Liu et al., 2018)	Humic acid-vitamin agar supplemented with cycloheximide (50 mg/L) and nalidixic acid (20 mg/L)	Formicary at Northeast Agriculture University, Harbin, China	Yu et al., 2013
*Streptomyces chiangmaiensis* sp. nov./ Streptomycetaceae	Apidae	East Asian stingless bee (*Tetragonilla collina*)	Whole tissue	Bees were surface-sterilized using a triple surface-sterilization technique and ground aseptically. Bacterial strains were isolated using the standard dilution-plate method on the isolation medium	Humic acid-vitamin agar supplemented with cycloheximide (50 mg/L) and nalidixic acid (20 mg/L)	Chiang Mai Province, northern Thailand	Promnuan et al., 2013
*Streptomyces lannensis* sp. nov./ Streptomycetaceae							

##### 4.2.1.2 Genus *Formica*

*Formica* is an ant genus comprised of honey dew feeding ants that are widely distributed in the Northern hemisphere (Borowiec et al., 2021). A comparative study of actinomycete abundance in ants by Zakalyukina et al. (2017) showed that actinomycetes are associated with *Formica cunicularia*, although less frequently than *Lasius niger*. Zakalyukina et al. (2017) reported the isolation of six *Streptomyces* spp. from adult worker *F. cunicularia*. Furthermore, novel compounds have also been reported from *Streptomyces* spp. associated with *Formica yessensis* (An et al., 2020; Du et al., 2020). The macrolides formicolide A and B were reported from *Streptomyces* sp. BA01 while formicin A-C were discovered from *Streptomyces* sp. SFA33 (Table 2).

**TABLE 2:** Compounds isolated from hymenopteran-associated actinomycetes in the last decade.

Compound(s)	Class	Producer strain	Source	Biological activity	References
Nocamycin V	Tetramic acid	*Amycolatopsis* sp.	*Trachymyrmex smithi*	Antibacterial	Hansen et al., 2022
Hamuramicin C	Macrolide	*Streptomyces* sp. *MBP16*	Gut of the wasp *Vespa crabro flavofasciata*	Anticancer	An et al., 2022
Colibrimycin A–C	Polyketides	*Streptomyces* sp. CS1 47	Unspecified Attine ant	Unknown	Prado-Alonso et al., 2022
Dentigerumycin F	Non-ribosomal peptide	*Pseudonocardia* sp. ICBG1122	Nest sample of the attine ant *Trachymyrmex* sp. collected in Brazil	Antifungal	Bae et al., 2021
Attinimicin	Non-ribosomal peptide	*Pseudonocardia* spp.	Nests of *Acromyrmex*, *Apterostigma* and *Trachymyrmex* species from Brazil	Antifungal	Fukuda et al., 2021
Formicolide A and B	Macrolides	*Streptomyces* sp. BA01	Gut of the wood ant *Formica yessensis*	Quinone reductase induction, antiangiogenic	An et al., 2020
Formicin A-C	Indenone Thioesters	*Streptomyces* sp. SFA33	Wood ant *F. yessensis*	Antiproliferative	Du et al., 2020
Kyamicin	Lanthipeptide	*Saccharopolyspora* sp. KY21	The ant *Tetraponera penzigi*	Weak antibacterial	Vikeli et al., 2020
Meliponamycin A and B	Hexadepsipeptides	*Streptomyces* sp. ICBG1318	The cuticle of the stingless bee *Melipona scutellaris*	Antibacterial	Menegatti et al., 2020
Strepantibin D and E	p-terphenyl glycosides	*Streptomyces* sp. N1510.2	Larva body of mud dauber wasp *Sceliphron madraspatanum*	Anticancer	Lu et al., 2019
Cornifronone	Cadinane-type sesquiterpene	*Streptomyces* sp. OC1611-8A	Body surface of the mason bee *Osmia cornifrons*	Hexokinase inhibition	Li et al., 2020
Strepantibin A–C	A and B are p-terphenyls while C is the first naturally occurring bisphenyltropone	*Streptomyces* sp. N1510.2	Larva of the mud-dauber wasp *S. madraspatanum*	Anticancer, Hexokinase inhibition	Song et al., 2019
Camporidine A and B	Polyketide alkaloids	*Streptomyces* sp. STA1	Gut of the carpenter ant *Camponotus kiusiuensis*	Anti-inflammatory, antimetastatic	Hong et al., 2019
Cyphomycin	Macrolide	*Streptomyces* sp. ISID311	The cuticle of the fungus-growing ant *Cyphomyrmex* sp.	Antifungal, antiprotozoan	Chevrette et al., 2019
Sipanmycin A and B	Macrocyclic lactam	*Streptomyces* sp. CS149	Leaf-cutting ants of the tribe Attini	Unknown	Malmierca et al., 2018
Cornifronin A and B	Polyketide	*Streptomyces* sp. OC1401	The body surface of the meson bee *Osmia cornifrons*	Antibacterial, antifungal	Wang et al., 2018
C_28_H_33_NO_5_	Spectinabilin derivative	*Streptomyces* sp. 1H-GS5	Head of the ant *Camponotus japonicus*	Cytotoxicity	Liu et al., 2016d
Selvamicin	Polyene	*Pseudonocardia LS1* and *LS2*	*Apterostigma* sp.	Antifungal	Van Arnam et al., 2016
9-methoxyrebeccamycin	Indolocarbazole	*Pseudonocardia BCI2*	*Apterostigma dentigerum* colonies	Antibacterial, anticancer	Van Arnam et al., 2015
Gerumycin A–C	Non-ribosomal peptide	*Pseudonocardia* sp. EC080625-04 and *Pseudonocardia* sp. HH130629-09	Cuticle of the attine ant *Apterostigma dentigerum*	Unknown	Sit et al., 2015
C_28_H_33_NO_6_	Spectinabilin derivative	*Streptomyces* sp. 1H-GS5	Head of ant *C. japonicus*	Cytotoxicity	Liu et al., 2015

##### 4.2.1.3 Genus *Lasius*

Ants of the genus *Lasius*, commonly known as black garden ants are also known to harbor actinomycetes. A study by Zakalyukina et al. (2014) showed that actinomycetes are equally as abundant in *Lasius niger* ant tissue as in their nests. Furthermore, Zakalyukina et al. (2017) isolated nine *Streptomyces* and one *Nocardia* from adult *Lasius niger* workers. Two more *Streptomyces* strains were reported from the same ant species in 2020 (Efimenko et al., 2020). In addition, four novel species of actinomycetes have been discovered from *Lasius* spp., including three from *Lasius fuliginosus* and one from *Lasius flavus* (Table 1).

##### 4.2.1.4 Genus *Polyrhachis*

Like carpenter ants, ants belonging to the genus *Polyrhachis* are members of the Camponotini tribe. Two novel species of actinomycetes, *Micromonospora polyrhachis* (Xiang et al., 2014) and *Streptomyces polyrhachii* (Yu et al., 2013), were isolated from the edible Chinese black ant (*Polyrhachis vicina*).

##### 4.2.1.5 Other genera

Actinomycetes have also been reported from formicine ant genera such as, *Oecophylla* (Hosmath and Timmappa, 2019), *Technomyrmex* (Diarra and Osborne-Naikatini, 2023), *Petalomyrmex* (Hanshew et al., 2015) and *Paratrechina* (Reyes and Cafaro, 2015; Matarrita-Carranza et al., 2017).

#### 4.2.2 Family Formicidae: subfamily Myrmicinae

##### 4.2.2.1 Genus *Allomerus*

Although much of the focus on actinomycetes within the subfamily Myrmicinae has been directed toward attine ants, actinomycetes have also been reported from non-attine members of the subfamily. The ant genus *Allomerus* is comprised of ants involved in a unique symbiosis with plants of the genera *Cordia* and *Hirtella* (Ruiz-González et al., 2011). *Allomerus* ants are carnivorous and mainly catch prey via entrapment. The ants build their traps along the stem and branches of their host plant using a combination of debris and fungi of the Order Chaetothyriales (Orivel et al., 2017). The fungus is believed to be deliberately farmed by the ants since different *Allomerus* species have been shown to associate with a monophyletic group of fungi. Nonetheless, till date, vertical transmission of the fungus has not been demonstrated (Seipke et al., 2013). Furthermore, Seipke et al. (2013) showed that *Allomerus* ants are associated with bioactive actinomycetes. The authors reported the isolation of three *Streptomyces* spp. and one *Amycolatopsis* sp. from *Allomerus decemarticulatus*, and three *Streptomyces* spp. from *Allomerus octoarticulatus*. Additionally, the authors demonstrated that some of the isolates inhibit pathogenic fungi derived from the ants’ nest. However, in a follow up study, the authors found a lack of evidence to support the claim that actinomycetes play a defensive role in *Allomerus* ants. This study utilized metagenomic analyses and found that actinomycetes occur very rarely on the cuticle of *Allomerus* ants in comparison to other groups such as *Erwinia* and *Serratia* species (Seipke et al., 2013). Therefore, there is insufficient evidence to support the idea that *Allomerus* ants recruit symbiotes of any kind to defend their fungal galleries; however, in the scenario where a defensive symbiote is likely, *Erwinia* and *Serratia* could be the culprits since members of both genera are known to produce antimicrobial compounds.

##### 4.2.2.2 Genus *Crematogaster*

Exploration of the ant *Crematogaster margaritae* which nests in domatia of the plant *Keetia hispida* led to the isolation of 121 Actinomycetota predominantly belonging to *Streptomyces*. Antifungal assay results showed that 3 out of the 121 isolates inhibit fungi *in vitro* (Hanshew et al., 2015). In a later study Matarrita-Carranza et al. (2017) reported the isolation of 10 actinomycetes from a member of the same genus (i.e., *C. longispina*).

##### 4.2.2.3 Genus *Messor*

Members of the genus *Messor*, commonly known as harvester ants, have also been explored with regards to association with actinomycetes. Wu et al. (2022) isolated two actinomycetes (*Brachybacterium phenoliresistens* and *Microbacterium* sp.) from *Messor orientalis* tissue and noted strong antifungal activity against plant pathogens. Furthermore, the actinomycete *Streptomyces globisporus* subsp. globisporus was reported from different colony components of *Messor structor* and found to be a new producer of albomycin (Zakalyukina et al., 2022b).

##### 4.2.2.4 Other genera

Actinomycetes have also been reported from two members of the genus *Pheidole* (Matarrita-Carranza et al., 2017), and *Tetramorium* (Diarra and Osborne-Naikatini, 2023).

#### 4.2.3 Family Formicidae: subfamily Dolichoderinae

Despite being among the most diverse ant subfamilies, there are few reports of actinomycetes in dolichoderine ants. Matarrita-Carranza et al. (2017) isolated eight actinomycetes from *Tapinoma ramulorum inrectum* while our recently work reports the isolation of six actinomycetes from the obligate plant-ant *Philidris nagasau* (Diarra and Osborne-Naikatini, 2023). A *Streptomyces antibioticus* strain isolated from *Tapinoma simrothi* was reported to produce a quercetin 3-O-glucoside derivative with broad-spectrum antimicrobial activity against human pathogenic bacteria and fungi (Nageh Sholkamy et al., 2020).

#### 4.2.4 Family Formicidae: subfamily Paraponerinae

This subfamily contains only one genus, *Paraponera* which inhabits tropical rainforests in Central and South America. *Paraponera clavata* (commonly known as the bullet ant) is the only extant member of the genus and samples of the species have been reported to contain actinomycetes (Matarrita-Carranza et al., 2017).

#### 4.2.5 Family Formicidae: subfamily Ponerinae

A study of the bacterial diversity of the gut of five ponerine species including *Dinoponera lucida*, *Pachycondyla curvinodis, Pachycondyla striata*, *Odontomachus brunneus* and *O. bauri* observed the presence of Actinomycetota in the gut microbiota all five species (Oliveira et al., 2016). A separate but similar study found that Actinomycetota are abundant in the gut microbiome of *O. monticola* and *Ectomomyrmex javanus* (Zheng et al., 2021). Moreover, Matarrita-Carranza et al. (2017) also reported the isolation of 25 actinomycetes, five of the isolates were found to exhibit antifungal activity against a *Metarhizium* sp., from four *Odontomachus* spp.

#### 4.2.6 Family Formicidae: subfamily Pseudomyrmecinae

With about 32 species residing in plant domatia, Pseudomyrmecinae is the most diverse plant-associated ant subfamily (Chomicki et al., 2015). Although members of the subfamily are predominantly tropical, some also occur in arid and subtropical regions (Ward, 2021). Pseudomyrmecinae contains about 230 species contained within only three genera, two of which have been reported to contain actinomycetes (Seipke et al., 2013; Chomicki et al., 2015; Hanshew et al., 2015).

##### 4.2.6.1 Genus *Pseudomyrmex*

Hanshew et al. (2015) obtained 60 Actinomycetota isolates from the ant-plant system of *Pseudomyrmex penetrator* and *Tachigali* sp., three of which displayed antifungal activity *in vitro*.

##### 4.2.6.2 Genus *Tetraponera*

Actinomycetes were first reported in this genus in a study by Seipke et al. (2013) which assessed the bacterial communities of plant-associated *Allomerus* spp. and *Tetraponera penzigi* using culture independent techniques. The findings of the study showed that Actinomycetota are abundant in the microbiome of *T. penzigi* and second only to Proteobacteria. Furthermore, the authors successfully isolated eight actinomycetes (5 *Streptomyces* spp. and 3 *Saccharopolyspora* spp.) from *T. penzigi*, with five isolates displaying antifungal activity. Nonetheless, the authors concluded that, like in *Allomerus* spp., actinomycetes are unlikely defensive symbiotes of *Tetraponera* (Seipke et al., 2013). Seipke and colleagues reported the discovery of a novel group of polyketide compounds, formicamycins, from one of the previously isolated *Streptomyces* strains (Qin et al., 2017). The producer strain, *Streptomyces formicae* KY5, was isolated from the domatium of an Acacia plant associated with *T. penzigi* and genome sequencing revealed that it contains 39 biosynthetic gene clusters (BGCs) (Seipke et al., 2013). Formicamycins exhibit potent antimicrobial activity against Gram-positive human pathogens including methicillin resistant *Staphylococcus aureus* (MRSA) and vancomycin resistant Enterococci (VRE) (Qin et al., 2017).

#### 4.2.7 Family Apidae: subfamily Apinae

##### 4.2.7.1 Genus *Apis*

Decade old studies have shown that honey bees harbor actinomycetes. Promnuan et al. (2009) reported the isolation of *Streptomyces*, *Nonomuraea* and *Nocardiopsis* from three honey bee species (*Apis mellifera*, *Apis cerana* and *Apis florea*) collected in Thailand. In a later study, the same authors described a novel actinomycete (*Actinomadura apis*) isolated from an *A. mellifera* hive in Thailand (Promnuan et al., 2011). In addition to hives, actinomycetes have been reported from worker bee tissue (Patil et al., 2010; Promnuan et al., 2021), bee larvae (Promnuan et al., 2021), honey (Promnuan et al., 2021), and pollen (Grubbs et al., 2021; Promnuan et al., 2021). While there is limited evidence to propose a symbiotic association between actinomycetes and honey bees, some bee isolates have been found to inhibit hive pathogens (Promnuan et al., 2009; Grubbs et al., 2021).

##### 4.2.7.2 Tribe Euglossa

Members of this genus are native to the neotropics and are commonly known as orchid bees. In their study of hymenopteran-associated actinomycetes, Matarrita-Carranza and colleagues sampled twelve colonies encompassing six species of the genus *Euglossa*. Out of the six studied species, actinomycetes were only present in two (i.e., *Euglossa heterosticta* and *Euglossa imperialis*).

##### 4.2.7.3 Tribe Meliponini

Meliponine bees (Tribe Meliponini, Family Apidae) form the largest grouping of eusocial bees with over 500 described species (Hrncir et al., 2016). The bees are commonly known as stingless bees due to their highly reduced stingers in comparison to other bees. Promnuan et al. (2009) reported the isolation of sixteen *Streptomyces* from two species of stingless bees, *Trigona laeviceps* (10 isolates) and *Trigona fuscobalteata* (6 isolates). Furthermore, the authors reported that six isolates were capable of inhibiting nest pathogens of the bees *in vitro*. In a 2019 study, seven actinomycetes (six *Streptomyces*, and one *Micromonospora*) were isolated from foraging and nurse *Melipona scutellaris* bees. Two isolates from the study, *Streptomyces* sp. ICBG1323 and *Micromonospora* sp. ICBG1321, yielded 15 compounds including lobophorins A, B, CR1, and K and ten anthracyclines (Rodríguez-Hernández et al., 2019). In the same year, Ngalimat et al. (2019) isolated a *Streptomyces kunmingensis* strain from the Malaysian stingless bee, *Heterotrigona itama*. Furthermore, two novel hexadepsipeptides were reported from a stingless-bee-associated *Streptomyces* and found to exhibit strong antibacterial activity against *Paenibacillus larvae* (Menegatti et al., 2020).

#### 4.2.8 Family Crabonidae: subfamily Craboninae

Besides beewolf wasps, actinomycetes have only been isolated from one crabonid genus (*Trypoxylon*) to our knowledge (Matarrita-Carranza et al., 2017).

#### 4.2.9 Family Pompilidae

To our knowledge, actinomycetes have only been reported from an unidentified member of this family, Pompilidae sp. A (Matarrita-Carranza et al., 2017).

#### 4.2.10 Family Sphecidae: subfamily Sceliphrinae

Sceliphrinae is a wasp subfamily that consists of solitary thread-waisted wasps with a cosmopolitan distribution. The subfamily contains six genera, two of which are known to build mud nests (Powell and Taylor, 2017). Nests are usually created by female mud-daubers to house the developing young and their food (paralyzed spiders or insects). The fact that nests are constructed out of mud and often contain paralyzed prey has led scientists to speculate a mechanism for nest hygiene involving actinomycetes (Poulsen et al., 2011; Kumar et al., 2012). Actinomycetes with bioactive properties have been isolated from two mud-dauber wasp species including yellow-black mud-dauber wasps (*Sceliphron caementarium*) and blue-black mud-dauber wasp (*Chalybion californicum*) (Poulsen et al., 2011). Additionally, the novel compound Sceliphrolactam was discovered from the fermentation broth of *Streptomyces flavogriseus* strain e122, isolated from *Sceliphron caementarium* (Oh et al., 2011). Nonetheless, there is a lack of evidence to suggest that actinomycetes are used by mud-dauber wasps as defensive symbiotes since neither symbiote specificity nor transmission has been demonstrated in the wasps.

#### 4.2.11 Family Vespidae: subfamily Polistinae

Polistinae consists of 25 genera and more than 900 species of eusocial vespid wasps (Gomes and Noll, 2009). Together with some members of the subfamilies Stenogastrinae, and Vespinae, members of Polistinae are social and construct paper nests (Höcherl et al., 2016).

##### 4.2.11.1 *Polistes*

The open-faced nature of paper wasp nests makes them prone to contamination by airborne bacterial and fungal spores leading some scientists to hypothesize defensive mechanisms. Madden et al. (2013), reported the isolation of thirty actinomycetes spanning three actinomycete genera (*Streptomyces*, *Micromonospora*, and *Actinoplanes*) from the nests of paper wasps. However, evidence to support a symbiotic relationship is weak as *P. dominulus* nests have been shown to be collection points for heavy metals originating from combustion engines and thus, may trap actinomycete spores in a similar manner (Urbini et al., 2006; Madden et al., 2013). Furthermore, Hoggard et al. (2011) showed that paper wasps produce cuticular antimicrobial compounds that could play vital roles in maintaining nest hygiene.

##### 4.2.11.2 *Polybia*

*Polybia* is a neotropical eusocial wasp genus comprising about 56 species (Prato et al., 2022). Nonetheless, actinomycetes have only been isolated from two species so far. These two species include *Polybia plebeja* and *P. occidentalis* from which 12 and 8 isolates were obtained, respectively (Matarrita-Carranza et al., 2017). Seven of the twenty isolates displayed *in vitro* antifungal activity against the wasp pathogen *Hirsutella citriformis* (Matarrita-Carranza et al., 2017). Furthermore, five macrocyclic antibiotics with antibacterial, antitumor and antiviral properties were identified from the extract of one of the isolates (i.e., *Streptomyces* sp. M54) in a later study (Matarrita-Carranza et al., 2021).

##### 4.2.11.3 Other genera

Twenty-two and twenty-four actinomycetes were isolated from members of *Agelaia*, *Metapolybia*, respectively, by Matarrita-Carranza et al. (2017).

## 5 Bioprospecting hymenopteran-associated actinomycetes

**FIGURE 3:**
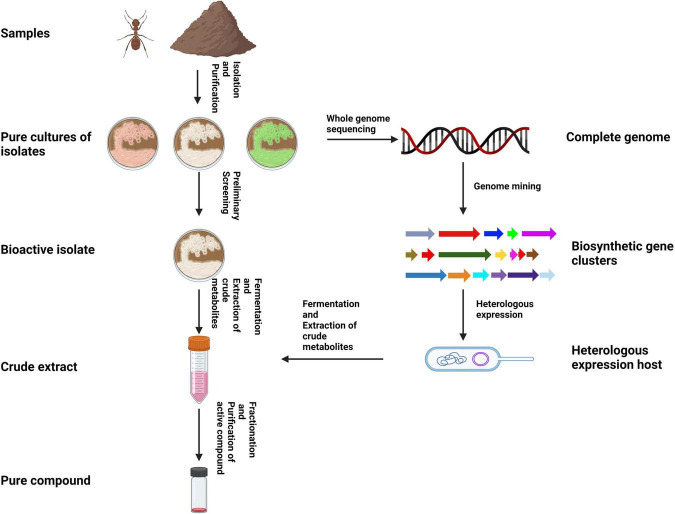
Illustration of the typical workflow for isolating and bioprospecting hymenopteran-associated actinomycetes using a traditional approach (created using BioRender.com).

Traditionally, actinomycetes have been isolated from samples using culture-dependent techniques. The appeal of such techniques is that the actinomycete of interest can be mass produced easily and the production of metabolites can be optimized readily. Most metabolite discoveries from actinomycetes have been conducted in this fashion (Figure 3). However, research suggests that less than 1% of actinomycetes have been identified till date because the vast majority of actinomycetes present in samples remain unculturable under conventional conditions (Subramani and Sipkema, 2019). The advent of high throughput culture-independent techniques such as metagenomics and metatranscriptomics in recent decades, have allowed researchers to directly study DNA from environmental samples (eDNA) to gain insights into the diversity, ecological roles, and biosynthetic potential of organisms (Seyedsayamdost et al., 2012).

### 5.1 Diversity of hymenopteran-associated actinomycetes

Hymenopteran-associated actinomycetes span several taxa including *Streptomyces* and rare genera. Like with most other environments, the most frequently isolated actinomycetes from hymenopteran samples are members of the genus *Streptomyces* (Matarrita-Carranza et al., 2017; Chevrette et al., 2019). In fact, among the novel actinomycete species discovered in the past decade, 58% (11 species) belong to genus *Streptomyces*. The remaining 42% of the novel species belong to rare actinomycete genera such as *Actinocorallia*, *Amycolatopsis*, *Micromonospora*, *Microbispora*, *Nocardia*, *Promicromonospora*, and *Virgisporangium* (Table 1).

Nonetheless, several other rare genera of actinomycetes have been isolated from hymenopteran samples including *Actinomadura* (Promnuan et al., 2009, 2021), *Actinoplanes* (Madden et al., 2013), *Kitasatospora Microbacterium* (Wu et al., 2022), *Nocardiopsis* (Promnuan et al., 2009; Kumar et al., 2012), *Nocardioides* (Hanshew et al., 2015), *Nonomuraea* (Promnuan et al., 2009), *Phytohabitans* (Wang et al., 2020), *Propionicimonas* (Zucchi et al., 2011), *Pseudonocardia* (Cafaro et al., 2011; Meirelles et al., 2014; Bruner-Montero et al., 2021), *Saccharopolyspora* (Kumar et al., 2012), *Saccharothrix* (Matarrita-Carranza et al., 2017)*, Streptosporangium* (Kumar et al., 2012; Matarrita-Carranza et al., 2017), *Thermoactinomycetes* (Kumar et al., 2012), *Tsukamurella* (Barke et al., 2010), *Verrucosispora* (Wang et al., 2020).

## 6 Bioactive potential of hymenopteran-associated actinomycetes

**FIGURE 4:**
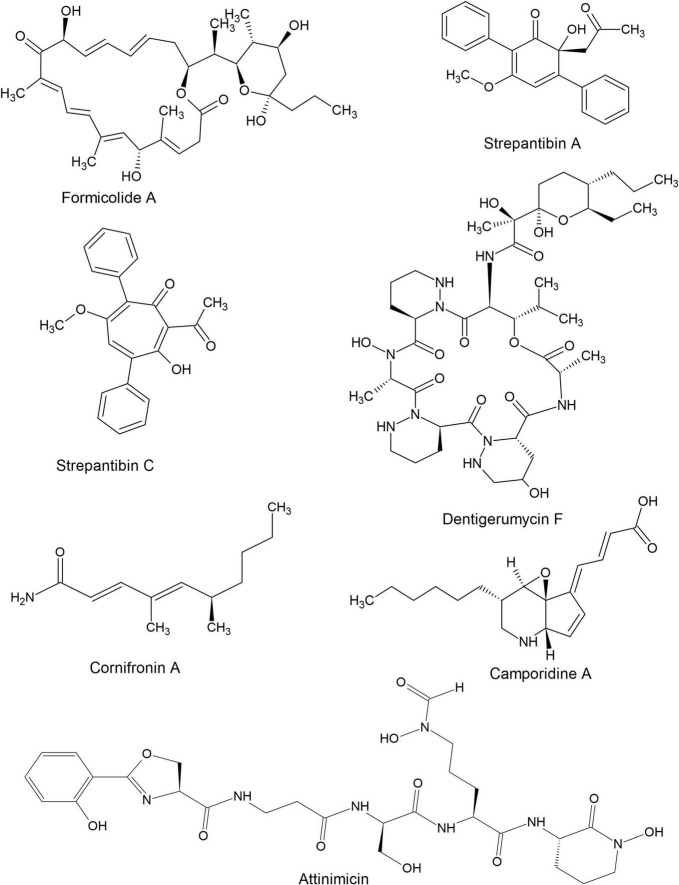
Structures of some bioactive compounds derived from hymenopteran-associated actinomycetes.

Hymenopteran-actinomycete associations represent an important source of natural products with potential applications in human medicine. Such complex symbiotic interactions have a unique potential in drug discovery due to pathogen pressure in hymenopteran insects which selects for association with actinomycete strains that produce efficacious antimicrobials. Chevrette et al. (2019) demonstrated that insect-associated *Streptomyces* strains exhibit greater inhibitory activity toward bacterial and fungal pathogens compared to soil and plant associated strains. Additionally, compounds isolated from hymenopteran-associated actinomycetes are not limited to inhibiting associated pathogens but also human pathogens thus, making them important in the fight against antimicrobial resistance. For example, dentigerumycin, the main antifungal compound produced by attine ant-associated *Pseudonocardia*, to combat *Escovopsis* infections. However, dentigerumycin also displays good inhibitory activity against amphotericin-resistant *Candida albicans* ATCC200955 (MIC- 1.1 μM) (Oh et al., 2009). At the same time, compounds derived from hymenopteran-actinomycete defensive mutualisms can display remarkable pathogen specificity. Dentigerumycin, for example, selectively inhibits *Escovopsis* pathogens of attine ants while causing no harm to the mutualistic fungus of the ants (Oh et al., 2009; Jiménez-Gómez et al., 2021). Finally, actinomycete associations with hymenopterans may select for compounds with low toxicity toward animal cells (Chevrette et al., 2019). Thus, making hymenopteran-associated actinomycetes an ideal source for natural products with therapeutic applications. Some novel compounds derived from hymenopteran-associated actinomycetes are shown in Figure 4.

### 6.1 Diversity of bioactive compounds isolated from hymenopteran-associated actinomycetes

Hymenopteran- associated actinomycetes are known to produce a wide range of bioactive natural products ranging from small to large and simple to complex (Figure 4). Compounds reported from hymenopteran-associated actinomycetes include polyketides (Qin et al., 2017; Prado-Alonso et al., 2022), macrolides (Van Arnam et al., 2016; Batey et al., 2020; Ortega et al., 2021; An et al., 2022), macrolactams (Oh et al., 2011; Beemelmanns et al., 2017), non-ribosomal peptides (Bae et al., 2021; Fukuda et al., 2021), ribosomally synthesized and post-translationally modified peptides (Vikeli et al., 2020), sesquiterpenes (Li et al., 2020), p-terphenyls, polyenes (Barke et al., 2010) and monohydroxypyridines among others. In the last decade, thirty-five compounds, shown in Table 2, were discovered from hymenopteran-associated actinomycetes. The genus *Streptomyces* stands out as the primary reservoir of hymenopteran-associated actinomycete-derived natural products, as evidenced by the discovery of twenty-six novel compounds from it (Table 2). Rare actinomycetes on the other hand, accounted for five novel compounds in the last decade including three compounds discovered from *Pseudonocardia* spp. (attinimicin, 9-methoxyrebeccamycin, and dentigerumycin F), one each from *Amycolatopsis* (nocamycin V) and *Saccharopolyspora* (kyamycin). Ants yielded 14 out of 19 actinomycete producer strains of the novel compounds while bees and wasps accounted for 3 and 2 producer strains, respectively. The biological properties of the novel compounds include inhibitory activities such as antimicrobial, anticancer, antiangiogenic, anti-inflammatory, antimetastatic antiproliferative, cytotoxic, hexokinase inhibition and quinone reductase promotion *in vitro* (Table 2).

## 7 Concluding remarks

Actinomycetes can be readily isolated from a wide range of hymenopteran insects but symbiotic relationships have so far only been established in attine ants and beewolf wasps. In both attine ants and beewolf wasps, symbiotic actinomycetes primarily perform defensive functions, thus making them uniquely suited to produce bioactive compounds. However, the exploration of hymenopteran-associated actinomycetes for natural product discovery, like with free-living actinomycetes, is challenged by factors such as low cultivability of microorganisms and the cryptic state of many BGCs under conventional conditions. While several methods have been developed to circumvent these issues, applications of these methods in the study of hymenopteran-associated actinomycetes are limited. Nonetheless, significant progress has been made in the past decade and research has yielded several novel strains of hymenopteran-associated actinomycetes with bioactive potential. Future studies should utilize sequence-based techniques such as metagenomics and metabolomics on hymenopterans to screen actinomycetes for bioactive compounds. Metabolomics and transcriptomics can also shed light on the origin of compounds produced by such actinomycetes, possible ecological roles of the compounds, and cues to express cryptic BGCs.

## Author contributions

UD: Conceptualization, Data curation, Formal analysis, Investigation, Methodology, Project administration, Writing – original draft. TO-N: Supervision, Writing – review & editing. RS: Conceptualization, Investigation, Supervision, Writing – original draft.

## In memoriam

Ramesh Subramani was involved in the conceptualization and planning of this project but tragically passed away in an accident before the completion.
